# Realtime Monitoring of Local Sweat Rate Kinetics during Constant-Load Exercise Using Perspiration-Meter with Airflow Compensation System

**DOI:** 10.3390/s22155473

**Published:** 2022-07-22

**Authors:** Hiroki Okawara, Tomonori Sawada, Daisuke Nakashima, Yuta Maeda, Shunsuke Minoji, Takashi Morisue, Yoshinori Katsumata, Morio Matsumoto, Masaya Nakamura, Takeo Nagura

**Affiliations:** 1 Department of Orthopaedic Surgery, Keio University School of Medicine, 35 Shinanomachi, Shinjuku-ku, Tokyo 160-8582, Japan; hiroki.okawara@keio.jp (H.O.); ksdyuta@keio.jp (Y.M.); shunsuke1301@keio.jp (S.M.); takashi.morisue@keio.jp (T.M.); morio@a5.keio.jp (M.M.); masa@keio.jp (M.N.); nagura@keio.jp (T.N.); 2 Institute for Integrated Sports Medicine, Keio University School of Medicine, 35 Shinanomachi, Shinjuku-ku, Tokyo 160-8582, Japan; tomonori-sawada@keio.jp; 3 Department of Cardiology, Keio University School of Medicine, 35 Shinanomachi, Shinjuku-ku, Tokyo 160-8582, Japan; 4 Department of Clinical Biomechanics, Keio University School of Medicine, 35 Shinanomachi, Shinjuku-ku, Tokyo 160-8582, Japan

**Keywords:** sweat rate, local sweat rate, sweating, perspiration, body temperature regulation, sports

## Abstract

Epidermal wearable sweat biomarker sensing technologies are likely affected by sweat rate because of the dilution effect and limited measurement methods. However, there is a dearth of reports on the local sweat rate (LSR) monitored in real-time during exercise. This explorative study investigated the feasibility of real-time LSR monitoring and clarified LSR kinetics on the forehead and upper arm during constant-load exercise using a perspiration meter with an airflow compensation system. This observational cross-sectional study included 18 recreationally trained males (mean age, 20.6 ± 0.8 years). LSR on the forehead and upper arm (mg/cm^2^/min) were measured during a constant-load exercise test at 25% of their pre-evaluated peak power until exhaustion. The LSR kinetics had two inflection points, with a gradual decrease in the incremental slope for each section. After the second flexion point, the LSR slope slightly decreased and was maintained until exhaustion. However, the degree of change varied among the participants. Although the ratio of forehead LSR to upper arm LSR tended to decrease gradually over time, there was little change in this ratio after a second flexion point of LSR in both. These findings suggest possible differences in LSR control between the forehead and upper arm during constant-load exercise to prolonged exhaustion.

## 1. Introduction

Recent progress in epidermal wearable chemical sensing technology allows the noninvasive acquisition of several significant biomarkers. Among the various detectable epidermal biomarkers, a considerable number of wearable bio-sensors are focused on sweat [1,2,3,4,5,6]. This is because sweat is readily accessible and contains important electrolytes, metabolites, amino acids, proteins, and hormones [7,8]. Particularly, sweat has the advantage of continuous profiling any alterations in body status during various forms of exercise, which is expected to contribute to a better understanding of unclear sports physiology—including real-time metabolism during swimming or ballgames—and be used in endurance evaluation [9,10] and determining fatigue status [11]. Unsurprisingly, the different types of sweat contain different concentrations of each component, likely due to sweat rate (SR). Sweat lactate is a byproduct of sweat gland metabolism associated with secretory sweat [7,8], and increased SR leads to the dilution of ammonia in sweat [12]. Although a high sweat production in highly intensive exercise was reported to provide a dilution effect on sweat lactate concentration [13], how exactly this affects the concentration is still unknown due to a lack of continuous data for the local sweat rate (LSR). Nevertheless, there has been speculation regarding the LSR kinetics during various exercise types or sports activities. Presumably, this is mainly because the elucidation of total body SR was more important in determining thermoregulation or dehydration status. Additionally, another possible reason is that the measurement methods of LSR dynamics had limited sensitivity and difficulty measuring the elevated amount of LSR produced during high-intensity exercise similar to a sports match [14,15,16,17,18,19]. Moreover, the traditional ventilated capsule only detects continuous LSR during exercise by adapting nitrogen gas or dehumidifying the gas using silica gel, making the measurement of LSR difficult in a sports setting due to the device’s cumbersome size. Recently, several studies attempted to elucidate LSR dynamics in response to mental stress by adding an airflow compensation system to the ventilated capsule [20]. This system requires no dry air to create a reference measurement environment and can optimize airflow according to the amount of perspiration present. While these features increase sensitivity, availability for increased LSR, reliability (coefficient of variation of 2%) [21], and portability—and are thus considered ready to be implemented in a sports setting—no report exists on the LSR dynamics when using the capsule during various exercise or sports activities. Therefore, we conducted this exploratory study to clarify the ability of the ventilated capsule method with an airflow compensation system to detect LSR dynamics during exercise. Further, we evaluated regional differences in these changes between two different parts of the body, which were selected to accommodate wearable bio-sensors.

## 2. Materials and Methods

### 2.1. Study Design

This cross-sectional study was performed at sports facilities between May and August 2020. The study investigated the kinetics of LSR during exercise using a perspiration meter with an airflow compensation system.

### 2.2. Participants

After providing written informed consent, 18 healthy males (age, 20.6 ± 0.9 years; height, 171.6 ± 5.8 cm; body mass, 63.3 ± 9.1 kg) with regular exercise habits, including basketball, soccer, tennis, handball, swimming, and ice hockey (2 to 3 h per day, more than three times per week). Individuals with orthopedic, metabolic, cardiac, respiratory, psychiatric abnormalities, or a smoking habit were excluded. The participants were asked to refrain from consuming any food and caffeine for 3 [22] and 12 h, respectively [23], before the exercise test. This was to ensure that no external factors affected participants’ endurance performance. Participants were also encouraged to be well hydrated before the test and allowed to drink water freely during the exercise test. All applicable institutional and governmental regulations concerning the ethical guidelines for studies involving human volunteers were followed over the course of this research. The study protocol was conducted in compliance with ethical guidelines for medical and health research involving human participants and was approved by the P-One Clinic Ethical Committee and our institute’s Ethics Committee (Approval no. 20190357).

### 2.3. Procedures

All participants’ body composition was evaluated using a multi-frequency segmental body composition analyzer (MC-780A-N, TANITA Co., Ltd., Tokyo, Japan) before the test.

The exercise test was based on the peak power calculated with the Wingate test equation with an applied resistance of 11% of participants’ body weight performed on another day [24]. All participants underwent a constant-load exercise test at 25% peak power until exhaustion. Exhaustion was defined as “an unsustainable state for pedaling at over 70 revolutions per minute (rpm)”. The cadence rate of pedaling was set at 70–80 rpm. The target constant workload was gradually reached in steps of 20 W every 30 s to avoid sudden strain after a one-minute warm-up at 50 W [25].

During the constant-load exercise test, LSRs were measured on the upper arm (aLSR) and forehead (hLSR) using a ventilated capsule-type perspiration meter with an airflow compensation system (SKN-2000M; SKINOS Co., Ltd., Nagano, Japan). This instrument consisted of a capsule (φ, 20 × 20 mm), two absolute humidity sensors, a humidifier, and an amplifier and air pump. The instrument had a measuring range of 0–4.0 mg/cm^2^/min and measurement accuracy of 5%. This instrument controls the airflow rate supplied to the capsule to keep a fixed flow rate of air with known humidity supplied to the capsule. This moves the air/gas that absorbed the evaporated water from the skin through and out of the capsule at a constant rate. The heat and humidity of the effluent gas are measured, obtaining the humidity difference, and allowing the SR to be calculated. Therefore, this system not only enables the detection of a wider range of values and immediate changes in LSR but is also highly portable. The data for each LSR were recorded every second.

### 2.4. Statistical Analysis

Time to exhaustion (TE) was defined as “the time (minutes) from the start of the constant-load exercise until exhaustion”. Metabolic heat production in each participant was calculated in relation to external work (W) per participant’s weight (kg). The onset of each flexion point of the LSR was defined as “the point at which each parameter increased markedly above the equilibration level (baseline)”. The LSR flexion point was based on graphical plots analyzed by three researchers, with reference to a previous report [26] and change finder scores. Change finder scores were calculated by a change finder algorithm [27], which consisted of two-step learning with a sequentially discounting autoregressive model to accurately distinguish between outliers and change points. In the pedaling exercise, the mean and peak LSRs were calculated in each section, segmented by two inflection points from the onset of sweating (OS) until exhaustion. Based on the results of the Shapiro–Wilk test, either the Wilcoxon or paired *t*-test was used to compare the hLSR and the aLSR, while effect size was calculated as an r-value using the Z- or t-value [28]. Similarly, repeated analysis of variance or the Friedman test with Bonferroni correction was used to compare LSR data in each section, and the effect size was calculated as η^2^ or partial η^2^. The regional difference between the hLSR and the aLSR was defined as “the hLSR divided by aLSR”. Data were analyzed using IBM SPSS Statistics, version 26.0 (IBM Corp., Endicott, NY, USA), with statistical significance set at *p* < 0.05.

## 3. Results

### 3.1. Kinetics of Local Sweat Rate

The characteristics of all participants who completed the exercise test are shown in Table 1 (individual data are shown in Table S1).

**Figure 1:**
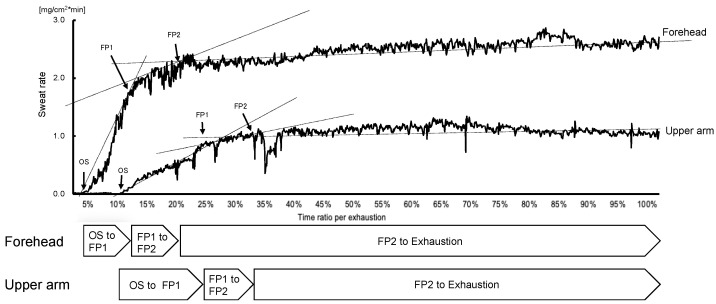
Representative data of the local sweat rate during constant-load exercise until exhaustion. This figure shows a representative graph of the local sweat rate over time from the start of the constant-load pedaling exercise to the end of the exercise (until exhaustion). After the onset of sweating, the sweat rate rapidly increases (OS to FP1), followed by a lower slope of the increment (FP1 to FP2), and finally, a state of slight decrease (FP2 to exhaustion), which is characteristic of the local sweat rate at the forehead. Abbreviations: FP, flexion point: OS, onset of sweating.

Exercise hLSR data could be obtained for 15 participants, excluding 2 participants whose LSR exceeded the measurable range and 1 participant who experienced capsule floating because of excessive sweating. Exercise aLSR data could be obtained in 17 participants, excluding 1 participant who experienced capsule floating. According to representative LSR data, during constant-load exercise to exhaustion, both hLSR and aLSR showed a rapid initial increase from OS in almost all participants. The incremental LSR slope then increased from the first flexion point (FP1) before flattening at the second flexion point (FP2). Lastly, the slope then slightly decreases until exhaustion (Figure 1).

**Figure 2:**
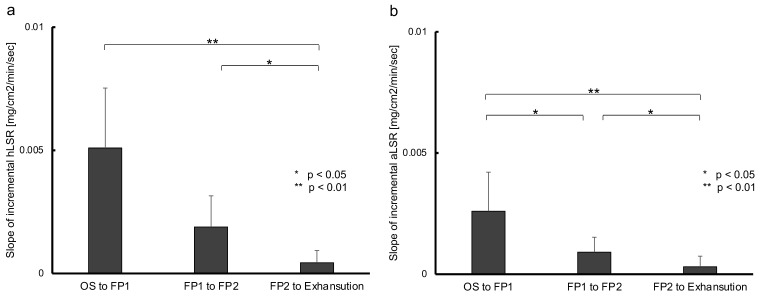
Change in the slope of incremental local sweat rate in the duration segmented by two inflection points during pedaling exercise. This figure shows the change in the slope of incremental local sweat rate from the onset of sweating to exhaustion in constant-load exercise. Each duration was divided into two inflection points of sweat rate over time. (**a**): Significantly gradual decreasing sweat rate measured at the forehead was detected over time using the Friedman test (*p* < 0.01, partial η^2^ = 0.79) and the Bonferroni method as the post-hoc test (OS to FP1 vs. FP1 to FP2, *p* = 0.07; FP1 to FP2 vs. FP2 to exhaustion, *p* = 0.014). (**b**): Significantly gradual decreasing sweat rate measured at the upper arm was detected over time using repeated-ANOVA (*p* < 0.01, η^2^ = 1.00) and the Bonferroni method (OS to FP1 vs. FP1 to FP2, *p* = 0.014; FP1 to FP2 vs. FP2 to exhaustion, *p* = 0.014). OS, onset of sweating; FP1, first flexion point; FP2, second flexion point; hLSR, local sweat rate measured at forehead; aLSR, local sweat rate measured at upper arm.

For both LSRs, the incremental LSR slope in each of the periods, from OS to FP1 (LSRslope [OS-FP1]), FP1 to FP2 (LSRslope [FP1-FP2]), and FP2 to exhaustion (LSRslope [FP2-Exh]), was calculated by dividing the difference between the LSR at the beginning and end of each period by the time of each period. Those slopes were decreased significantly over time (hLSR, *p* < 0.01, partial η^2^ = 0.79; aLSR, *p* < 0.01, η^2^ = 1.00; Figure 2). One participant showed unique kinetics for hLSR and aLSR, which had more than two flexion points and decremental LSR following the peak (Figure S1). Notably, this participant had the highest body fat ratio (24.5%) and the lowest body water ratio (49.9%) of the 18 participants (Table S1). The data of this participant was excluded from further analysis.

### 3.2. Regional Difference in Sweat Rate

Data of participants who had two inflection points for LSR for each of the three SR sections (separated by the two inflection points) of each LSR (from OS to exhaustion) are shown in Table 2. Each statistical method adopted was demonstrated in Table 2 per comparison. The mean and peak LSRs significantly increased for each section (mean hLSR, *p* < 0.01, partial η^2^ = 0.88; peak hLSR, *p* < 0.01, partial η^2^ = 0.76; mean aLSR, *p* < 0.01, η^2^ = 1.00; peak aLSR, *p* < 0.01, η^2^ = 0.88). Additionally, there were significant regional differences in LSRs for each section, with moderate to large effect sizes (hLSR > aLSR, r = 0.73–0.93; Table 2).

**Figure 3:**
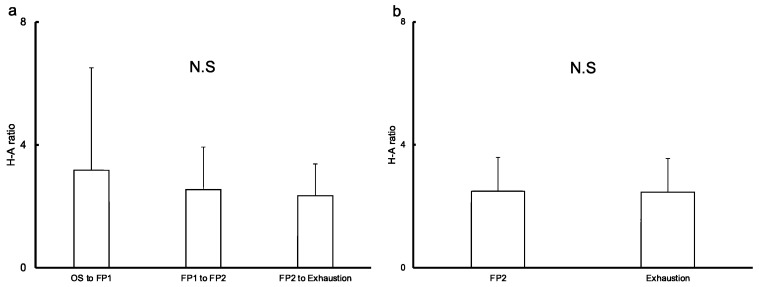
Regional difference in mean local sweat rate in each duration based on two flexion points of sweat rate. This graph shows the ratio of the mean local sweat rate on the forehead to that on the upper arm in each section, segmented by two inflection points, from the onset of sweating until exhaustion. The ratio tended to decrease over time, although there was no significant difference (**a**), while a slight change was observed from the second flexion point to exhaustion. There was little change of the ratio of the time from exercise start to each time point detected on the forehead to that on the upper arm (**b**). Abbreviations: FP, flexion point; OS, onset of sweating; H-A ratio, the ratio of mean local sweat rate on the forehead to that on the upper arm, N.S, not significant.

The mean hLSR to mean aLSR ratio in each section tended to decrease over time, although not significantly (OS to FP1, 3.18 ± 3.33; FP1 to FP2, 2.55 ± 1.38; FP2 to exhaustion, 2.35 ± 1.03). However, there was little change in this ratio from the time both the hLSR and aLSR reached FP2 to exhaustion (FP2, 2.49 ± 1.10; exhaustion, 2.46 ± 1.09; Figure 3).

## 4. Discussion

We evaluated LSR on the forehead and upper arm from OS to exhaustion during the constant-load pedaling exercise using a ventilated capsule-type perspiration meter with an airflow compensation system. Our findings provide information on the capability of a sweat loss measuring device to detect the kinetics of a large amount of local sweat continuously. Our results showed two inflection points, with a gradual decrease in the incremental slope for each section. After FP2, the negative change in LSR continued until exhaustion, despite variability in the degree of change among participants. Additionally, while regional differences between the hLSR and aLSR tended to decrease over time, there was little change in the difference in the two from FP2 to exhaustion.

LSR during constant-load exercise showed different features for each section, which was divided by two inflection points (Figure 1). The rapid increase from OS to FP1 in our results coincides with previous reports [29,30]. Similarly, it has already been reported that LSR reaches a steady state following an initial increase [31,32]. However, the existence of two inflection points during constant-load exercise has never been reported. Particularly, the kinetics of LSR during exercise is still unknown. Therefore, our results demonstrate a novel finding of two inflection points in LSR kinetics—the latter being followed by a state of slight decrease until exhaustion. However, the degree of changes in LSR varied among participants in the period from FP2 to exhaustion. It is possible that these present findings with LSR kinetics will contribute to interpreting the result obtained via real-monitoring biomarkers in sweat, which is beneficial for the evaluation of body status during exercise. The reason for this is that several analytes have the possibility to provide helpful information about body status by detecting the flexion point in a continually obtained value during exercise [9,10,11,33]. Further research or application in sports settings to the monitoring of analytes in sweat during constant-load exercise needs to interpret obtained results on the premise of the existence of two flexion points in LSR kinetics. Moreover, the factor determining these two flexion points is unclear in our study. Further research to resolve this question is required for the progression of wearable biosensing technology targeting the analytes in sweat.

Our results showed little change in regional differences between hLSR and aLSR once both LSRs reached FP2 until exhaustion, albeit a trend of decremental difference over time from OS to exhaustion (Figure 3). Few studies have reported the change in regional differences in LSR over time during constant-load exercise. However, the decremental regional LSR difference in this study is consistent with some reports of a higher exercise intensity leading to lower regional differences in LSR [29,34]. These studies’ results were postulated to indicate that better evaporation is induced from a large segmental surface area-to-mass ratio and an increase in skin wetness in harder exercise [34]. Therefore, decreasing the difference in each LSR may provide better evaporation, allowing the whole-body SR to become steady even during constant-load exercise. For the application of epidermal wearable chemical sensing technology, where the epidermal wearable biosensor is attached is an important factor. As an example, wearable sensors for volatile organic compounds released through the skin are recommended to measure at external ears, which is a body site with less interference [35]. As regards wearable biosensors for analytes in sweat, the determination of attachment location should be taken into account, as shown in previous reports [7,8,12], as it affects the amount of perspiration. Considering the findings of this study and previous reports, it is recommended that the appropriate sensor location for measurement during constant-load exercise should be determined based on regional differences in local perspiration detected in short exercise or low-intensity exercise. Hence, the forehead is the preferable location when the population that is expected to sweat poorly, including patients with cardiac disease or on dialysis, is the target of analysis.

In this study, an individual with specific LSR kinetics was observed (Figure S1). This participant had the highest body fat and lowest body water ratio. However, it is difficult to determine the influence of these factors on LSR kinetics results since this was only a single case. However, it is possible that the abovementioned factors can affect LSR kinetics since the combination of hypohydration and high body fat has been reported to partially affect thermoregulation [36]. Hence, the homogeneity of participants in this study may have introduced limited results for only a limited subgroup also. Meanwhile, this homogeneity enhances the minimization of the influence of confounding factors on current results. Further research is needed to elucidate this finding.

There are several limitations to this study. First, we did not measure skin and body temperature, making it difficult to determine the degree of change in LSR increment during exercise. Second, we could not control the room temperature and humidity because we performed this observational study at sports facilities (24–26 °C, 45–65%), and this uncontrolled environment may have affected our results. However, the ambient conditions remained steady for each participant throughout the exercise tests.

## 5. Conclusions

In conclusion, ventilated capsule-type perspiration meters enable real-time monitoring of LSR data, including a considerable range of high-volume sweat during exercise. LSR had two inflection points during constant-load exercise and reached a point of slight decrease at FP2, which continued until exhaustion. However, the results of regional differences between hLSR and aLSR suggest differences in regional control of hLSR and aLSR. These findings may be helpful in determining the place where biosensors attach and interpret obtained results in further research or application in sports settings to the monitoring of analytes in sweat.

## Figures and Tables

**Table 1:** Characteristics and exercise data of study participants.

Variable	*n* = 18
Age (yr)	20.6 (0.9)
Height (m)	171.6 (5.8)
Weight (kg)	63.3 (9.1)
BMI (%)	21.4 (2.0)
Body fat ratio (%)	16.0 (4.3)
Lean body mass (kg)	52.9 (5.8)
Muscle mass (kg)	50.2 (5.5)
Total body water (kg)	36.7 (4.6)
Body water (%)	57.7 (4.6)
TE (s)	2080.8 (1271.1)
Pedaling load (W)	164.1 (39.5)

This table shows the characteristics and exercise data of all study participants. All values are shown as mean (SD). BMI, body mass index; TE, time to exhaustion.

**Table 2:** Difference in local sweat rate during constant-load exercise until exhaustion.

	Forehead	Upper Arm	r	*p*-Value
Time to point (s)				
Start to OS	270.9 (92.0)	321.5 (146.1)	0.52 ^§^	0.06
OS to FP1	266.6 (201.4)	299.6 (217.9)	0.52 ^§^	0.06
FP1 to FP2	662.0 (519.0)	756.4 (527.3)	0.26 ^§^	0.35
FP2 to exhaustion	1173.6 (989.6)	868.4 (794.2)	0.53 ^§^	0.06
Mean SR (mg/cm^2^/min)				
OS to FP1	0.66 (0.41) *^,†^	0.37 (0.28) *^,†^	0.73 ^§^	<0.01
FP1 to FP2	1.85 (0.60) ^†^	0.99 (0.47) ^†^	0.88 ^§^	<0.01
FP2 to exhaustion	2.37 (0.65)	1.29 (0.48)	0.88 ^§^	<0.01
Total	1.98 (0.54)	0.98 (0.37)	0.95 ^‖^	<0.01
Peak SR (mg/cm^2^/min)				
OS to FP1	1.35 (0.59) *^,†^	0.73 (0.48) *^,†^	0.80 ^§^	<0.01
FP1 to FP2	2.33 (0.70) ^‡^	1.32 (0.49) ^†^	0.91 ^‖^	<0.01
FP2 to exhaustion	2.72 (0.72)	1.51 (0.48)	0.93 ^‖^	<0.01
Total	2.72 (0.72)	1.50 (0.48)	0.93 ^‖^	<0.01

This table shows local sweat rate (LSR) data measured on the forehead and upper arm at each stage. Stages during pedaling exercise are segmented by two inflection points from the onset of sweating until exhaustion. Data are presented as mean (SD). The Wilcoxon test and paired *t*-test demonstrated significant local differences in mean and peak sweat rates in all periods with medium to high effect sizes. Moreover, mean and peak sweat rates significantly increased in each duration (forehead, *p* < 0.01 using repeated ANOVA test; upper arm: *p* < 0.01 using Friedman test). OS, onset of sweating; SR, sweat rate; FP1, first flexion point; FP2, second flexion point. * The Bonferroni method shows a significant difference between FP1 and FP2 (*p* < 0.01). ^†^ The Bonferroni method shows a significant difference between FP2 and exhaustion (*p* < 0.01), ^‡^ (*p* < 0.05), ^§^ Using Wilcoxon test, ^‖^ Using paired *t*-test.

## Data Availability

The data that support the findings of this study are available from the corresponding author upon reasonable request.

## Supplementary Materials

The following supporting information can be downloaded at: https://www.mdpi.com/article/10.3390/s22155473/s1, Figure S1: Representative data of sweat rate during constant-load exercise until exhaustion in the participant with more than two flexion points (participant no.14); Table S1: Individual characteristics and body composition data of study participants.
