# Supplementary material for: Realtime Monitoring of Local Sweat Rate Kinetics during Constant-Load Exercise Using Perspiration-Meter with Airflow Compensation System
Source: Sensors (Basel). 2022 Jul 22;22(15):5473. doi: 10.3390/s22155473 (PMC9331097; doi:10.3390/s22155473)

**Table S1.** Individual characteristics and body composition data of study participants

This table shows individual data of characteristics and body composition in 18 participants.

BMI, body mass index; Hprod, Heat production (External work per weight)

| No | Age [yr] | Height [cm] | Weight [kg] | BMI | Body Fat ratio [%] | Fat mass [kg] | Lean Body Mass [kg] | Muscle mass [kg] | Total Body Water [kg] | Body Water ratio [%] | Hprod [W/kg] |
|---|---|---|---|---|---|---|---|---|---|---|---|
| 1 | 21 | 168.8 | 57.3 | 20.1 | 12.0 | 6.9 | 50.4 | 47.8 | 35.4 | 61.8 | 1.78 |
| 2 | 20 | 170.2 | 59.1 | 20.4 | 14.0 | 8.3 | 50.8 | 48.2 | 34.6 | 58.5 | 2.84 |
| 3 | 21 | 174.9 | 60.9 | 19.9 | 16.6 | 10.1 | 50.8 | 48.2 | 33.9 | 55.7 | 2.70 |
| 4 | 20 | 163.1 | 47.1 | 17.7 | 7.5 | 3.5 | 43.6 | 41.3 | 30.8 | 65.4 | 2.71 |
| 5 | 19 | 169.6 | 64.7 | 22.5 | 18.1 | 11.7 | 53.0 | 50.2 | 35.1 | 54.3 | 2.62 |
| 6 | 20 | 168.1 | 55.4 | 19.6 | 12.9 | 7.1 | 48.3 | 45.8 | 33.2 | 59.9 | 3.40 |
| 7 | 20 | 164.5 | 57.4 | 21.2 | 21.0 | 12.1 | 45.3 | 42.9 | 29.2 | 50.9 | 1.62 |
| 8 | 21 | 165.1 | 48.8 | 17.9 | 10.8 | 5.3 | 43.5 | 41.2 | 30.0 | 61.5 | 2.53 |
| 9 | 22 | 180.1 | 80.4 | 24.8 | 20.4 | 16.4 | 64.0 | 60.7 | 46.2 | 57.5 | 3.28 |
| 10 | 21 | 170.3 | 64.1 | 22.1 | 16.3 | 10.4 | 53.7 | 50.9 | 37.8 | 50.0 | 2.38 |
| 11 | 21 | 181.9 | 71.8 | 21.7 | 19.4 | 13.9 | 57.9 | 54.9 | 39.2 | 54.6 | 2.82 |
| 12 | 21 | 166.1 | 54.9 | 19.9 | 11.0 | 6.0 | 48.9 | 46.3 | 34.3 | 62.5 | 2.86 |
| 13 | 21 | 181.7 | 75.9 | 23.0 | 20.1 | 15.3 | 60.6 | 57.5 | 41.2 | 54.3 | 2.12 |
| 14 | 21 | 173.6 | 71.4 | 23.7 | 24.8 | 17.7 | 53.7 | 50.9 | 35.6 | 49.9 | 2.24 |
| 15 | 20 | 175.8 | 70.8 | 22.9 | 16.8 | 11.9 | 58.9 | 55.8 | 41.7 | 58.9 | 2.24 |
| 16 | 19 | 170.2 | 67.2 | 23.2 | 15.9 | 10.7 | 56.5 | 53.6 | 40.5 | 60.3 | 2.77 |
| 17 | 22 | 176.8 | 68.8 | 22.0 | 16.4 | 11.3 | 57.5 | 54.5 | 40.5 | 58.9 | 2.45 |
| 18 | 21 | 168.2 | 64.2 | 22.7 | 14.0 | 9.0 | 55.2 | 52.3 | 40.7 | 63.4 | 3.23 |

[illegible]

**Figure S1**. Representative data of sweat rate during constant-load exercise until exhaustion in the participant with more than two flexion point (participant no.14)

This figure shows sweat rate data in participant who demonstrated specifical kinetics of sweat rate during constant-load exercise until exhaustion. This participant had more than two flexion points and decremental local sweat rate.

OS, onset of sweating; FP, flexion point

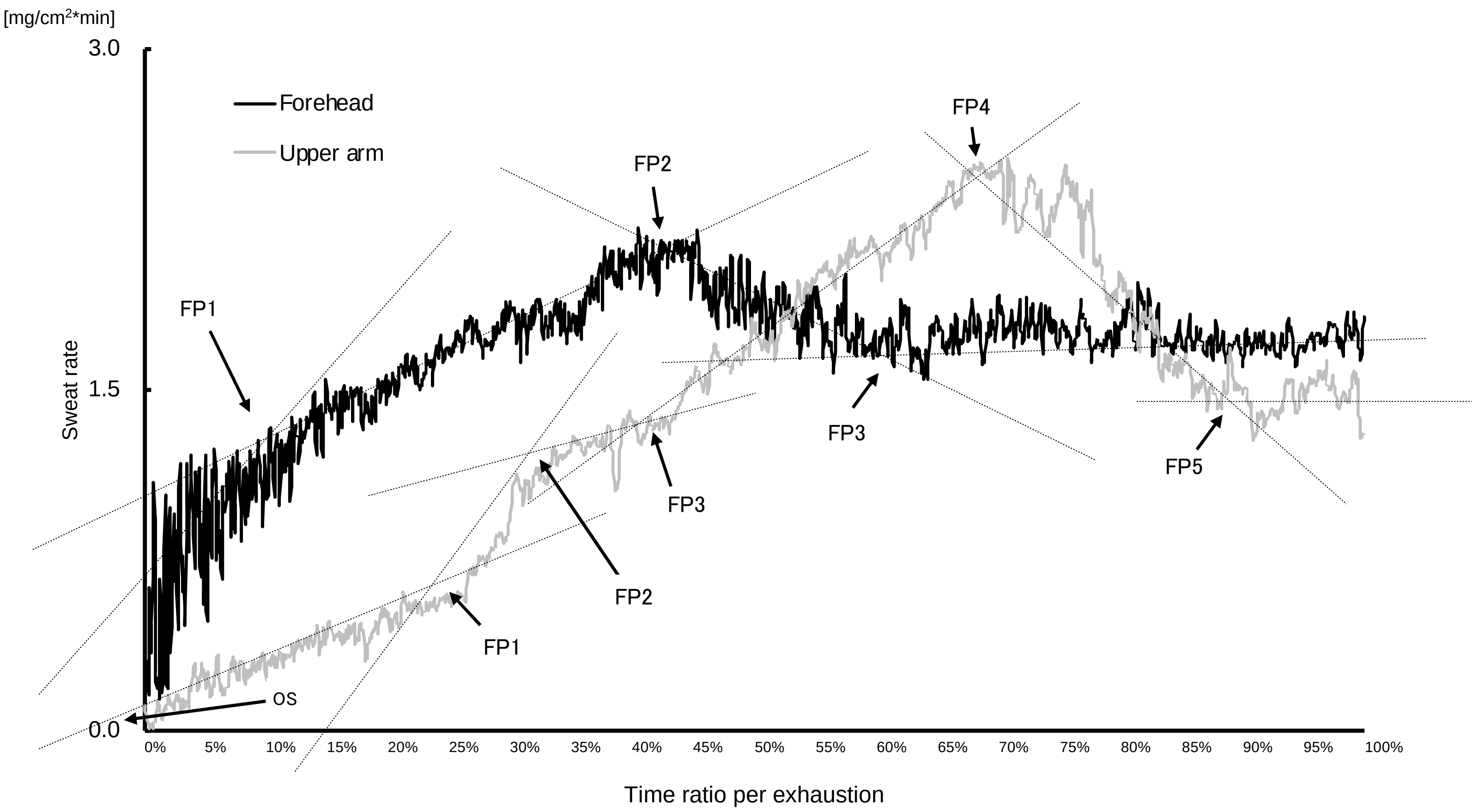

Supplement: Supplementary file 1 [file sensors-22-05473-s001.zip › sensors-1798502-supplementary.pdf]
